# Government Health Expenditure and Maternal Mortality: The Moderating Role of External Debt

**DOI:** 10.3390/healthcare12202030

**Published:** 2024-10-12

**Authors:** Gildas Dohba Dinga, Gisele Mah, Teboho Mosikari

**Affiliations:** Department of Economics, North West University, Mafikeng 2735, South Africa; gisele.mah@nwu.ac.za (G.M.); teboho.mosikari@nwu.ac.za (T.M.)

**Keywords:** government health expenditure, external debt, maternal mortality, moderation, augmented mean group, causality

## Abstract

Background/Objectives: The impact of government health spending and external debt on maternal mortality has been the subject of ongoing theoretical and empirical discussions. However, this relationship has remained controversial with no perspective on the moderating role of external debt on the government’s health expenditure and maternal mortality link. This study examines the moderating effect of external debt on the government’s health expenditure and maternal mortality relation using data from 13 Southern African economies spanning from 2000 to 2022. Methods: We employed the augmented mean group, the dynamic common correlation effect mean group, and the Driscoll–Kraay and Granger causality techniques to attain the study’s objective. Results: The outcome revealed that government health expenditure and external debt reduce maternal mortality in the Southern African Development Community (SADC) region. Equally, the magnitude of government health spending is moderated by external debt. The results revealed a bidirectional relation amidst maternal mortality and government health expenditure, and maternal mortality and external debt. Conclusions: The study recommends that policymakers within the SADC zone should avoid austerity measures and encourage expansionary measures in terms of spending, and the contraction of debt for capital investment in the health sector. This will enhance the delivery of health services within the zone and equally reduce the rate of maternal mortality that is still a major health concern within the sub-region.

## 1. Introduction

The global urge to ensure universal health for all remains a fundamental human right in the world today. The Sustainable Development Goal (SDG), goal number 3, highlights the essence of good and quality health for all. However, poor health remains one of the main worries in the world today generally, especially in developing countries [1,2,3]. Among health-related problems, maternal and neonatal morbidity and mortality remain vital issues that are disturbing the world globally, and sub-Saharan Africa, in particular [2,3]. Globally, developing nations account for 99 percent of all maternal and newborn fatalities and these fatalities are not distributed equally around the world [4,5]. Even if the prevalence is high in developing nations, the situation might be deemed dire because 66% of maternal deaths worldwide occur in the sub-Saharan African (SSA) region [5]. Cognizant of the United Nations SDG 2030, the survival target for maternal mortality (below 70 per 100,000 live births for maternal mortality and no country going beyond 140), the existence of an equity-specific target for the different mortality indicators for developing countries remains lacking, with relatively few developing countries, especially those of SSA, having sought to establish and or use such targets [6].

Furthermore, recent extant statistics demonstrates that, for the period of 2000 to 2017, maternal mortality decreased by 2.9 percent, neonatal mortality declined by 2.9 percent between the period of 2000 and 2019, and still birthrate decreased by 2.3 percent within the same period [6]. With the current global progress, however, SSA remains lagging behind in all regions, especially in maternal mortality [7]. Ref. [7] further highlighted that, although maternal mortality has reduced in Africa, only the northern Africa region is close to the SDG’s target. The remaining regions are still far from reaching the target. With the high prevalence of mortality amidst a current globalized world, with the required capital to meet the demand for different health medical facilities like the construction of hospitals, the purchase of updated medical equipment, and the training of quality medical personnel, a key question remains—namely, the role played by state financial mobilization and external sources of finance like external debt in boosting health status within a developing region like SSA and SADC, in particular.

Domestic efforts through state health expenditure have been identified within the extant literature as a key driver of economic performance and health outcomes like maternal mortality [8,9,10,11,12]. Theoretical arguments have been put forth within the framework of the health demand theory and the human capital theory. Ref. [13], the health demand theory, posits that people invest in their health by using several methods, such as healthcare utilization, to reach a desired state of health. Augmented health expenditure can be perceived as health capital investment for individuals (particularly, pregnant women) which can translate to better health outcomes like a reduction in maternal mortality. Deductively, channeling more finances into the health system, especially in developing economies like those of the SADC zone, can lead to an enhancement in health facilities, which will subsequently translate to a better health status for the population at large, inclusive of maternal health. Furthermore, Ref. [14] posits, within the framework of the human capital theory, that investment in healthcare enhances human capital development. This implies that government health expenditure is seen as building a healthy population, inclusive of women of reproductive age. Hence, improving maternal health through health expenditure, be it from domestic state capital mobilization or from external debt, will improve human capital, which subsequently leads to growth and development within economies.

The empirical arguments have remained conflicting within the past decades. Ref. [9] empirically demonstrated, from a panel of 46 African economies, that an increase in health spending reduces maternal mortality. Similarly, [10] showed, using the generalized method of moment (GMM) and ordinary least squares method, within Asian economies from 2000 to 2019, that health expenditure increases life expectancy and reduces maternal mortality. Using data from Asian economies, Ref. [15] showed empirically that health expenditure increased maternal mortality during the period of 2000 to 2017. A decrease in health spending has equally been shown to increase maternal mortality within 24 European Union economies [12]. In examining maternal mortality within the framework of a cross-sectional study for global health using 439 indicators, Ref. [16] identified the private sector and trade, education, economic policy and debt, infrastructure investment, and health finance as vital in shaping maternal mortality. Although controversial in terms of its effect on maternal mortality, public spending can be observed as a key driver of maternal deaths both within and without developing economies. Ref. [17] examined the determinants of health expenditures in Southern African economies using the GMM technique for the period of 2005 to 2019. Their main outcome shows, among others, that health expenditure was positive but insignificant, whereas HIV infection, GDP per capita, fertility, education, and institutional quality were all equally recognized as determinants. Refs. [11,18] have all established empirical evidence that health spending reduces maternal mortality. A key point to emphasize is that, if advanced economies can boost sufficient funds to meet their healthcare demands, the tales of developing regions like the SADC region are controversial with limited funds available to public and private actors to cover healthcare financing. These economies are forced to source extra funds through different sources like external debt.

Debt has recently become topical in the maternal mortality literature. External debt may help lower maternal mortality by funding health initiatives and enhancing the healthcare infrastructure. Conversely, high levels of external debt can result in increasing debt payment expenses, which can syphon funds from important public services, like healthcare. Theoretical arguments based on the dependency theory and economic hardship theory all point to the negative impact of high exterior debt on economic progress and an increase in maternal mortality. A study by [19] looks at the connection between the government’s health spending and debt relief under a heavily indebted poor country (HIPC) for 487 African economies from 2000 to 2019. The findings show that involvement in the HIPC project is linked to maternal mortality and the outcome was significant. An increase in state health spending as a percentage of GDP also significantly decreases the lifetime risk of maternal death and the maternal mortality ratio. Ref. [20] employed the Hechman Model on four points’ (1990, 1995, 2000, and 2005) cross-sectional data for Africa and established, among others, that, maternal mortality rates are typically higher in sub-Saharan African countries that receive structural adjustment loans from the International Monetary Fund than in those that do not. Ref. [21] concluded from a panel of SSA economies that structural adjustment lending by the World Bank increases maternal mortality while investment lending reduces maternal mortality. Ref. [22] makes use of cross-sectional data from 65 poor nations to examine women and the health of non-governmental organizations (NGOs) on maternal mortality. They concluded that higher rates of debt servicing, structural adjustment, and investment by multinational corporations are associated with higher rates of maternal mortality.

**Figure 1:**
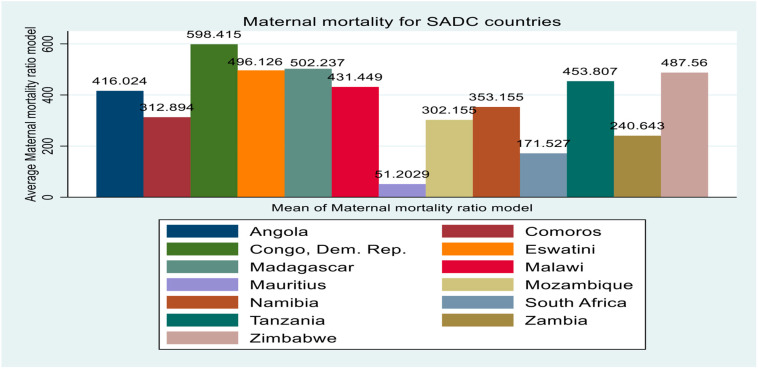
Average maternal mortality for SADC countries.

**Figure 2:**
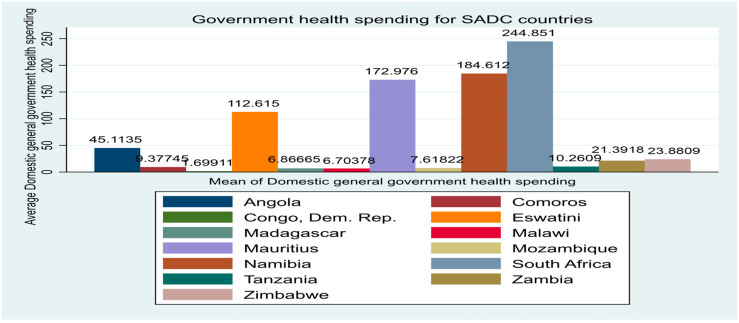
Average government health expenditure for SADC countries.

**Figure 3:**
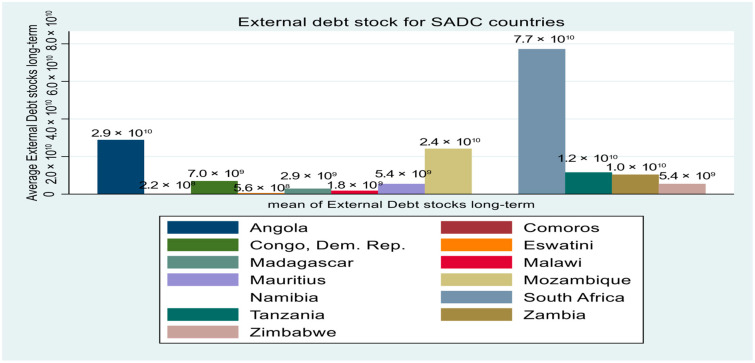
Average external debt stock for SADC countries.

With extant stylized facts compared to other SSA sub-regions, the SADC region has observed a relative fall in the rate of maternal mortality on average in the past decades [7]. However, mortality remains high in some economies. Figure 1 shows the mean mortality rate per 1,000,000 live births for 13 SADC economies from 2000 to 2022. It is observed that countries like the Democratic Republic of Congo, Madagascar, Eswatini, and Zimbabwe demonstrate the highest maternal mortality of above 480, whereas countries like South Africa and Mauritius show an average mortality of less than 200. Comparatively, during the same period, the average government health expenditure per capita for these 13 economies, reported in Figure 2, shows that South Africa, Namibia, and Mauritius have the highest expenditure of above 170 on average. Countries like the Democratic Republic of Congo and Madagascar show the lowest health expenditure. In the same vein, the average external debt disparity reported in Figure 3 demonstrates that economies like South Africa, Angola, and Mozambique have the highest instances.

The diverse literature reviewed demonstrates that the public expenditure and mortality nexus has been examined globally, and the moderating role of external debt is seen as lacking. The debt and maternal mortality nexus has generally been tilted towards debt relief initiatives with little consideration of the financial gaps these debts can fill within domestic developing economies, which can go a long way to boosting healthcare financing. The controversial stylized facts reported equally demonstrate some insights into the common trend between maternal mortality, public expenditure, and external debt in terms of peak and floor economies. Based on the stylized facts reviewed, and the theoretical and empirical literature discussed, it becomes imperative that we question the role of government expenditure and foreign debt in shaping the trend of maternal mortality within the SADC sub-regions. This study addresses this question by not just examining public expenditure and debt on maternal mortality, but equally examining the moderating effect which makes it stand out within the existing literature. This current study equally employs second-generation estimation methods that take into consideration problems like cross-sectional dependence, which leads to a more efficient outcome.

The other portions of this study are structured as follows: Section 2 provides the study’s materials and procedures; Section 3 displays the results of the empirical analyses; and Section 5 offers conclusions and recommendations for policy.

## 2. Methods and Materials

### 2.1. Description of Data Sources and Variables

To achieve the goal of this research, which is to investigate how health expenditure affects maternal mortality in the SADC sub-region and how foreign debt modifies this relationship, the study uses data from the World Development Indicator [23]. The dataset encompasses 13 SADC (Angola, Tanzania, Madagascar, Comoros, Zambia, Dem. Rep. of Congo, Eswatini, Malawi, Namibia, Mauritius, Mozambique, South Africa, and Zimbabwe) economies spanning the period of 2000 to 2022. The study period and number of SADC economies were selected subject to the availability of data and equally based on the fact that the Millennium Development Goals were adopted in the year 2000, with one of the key targets being health outcome enhancement [24]. The measurement of maternal mortality is represented by the estimated maternal mortality ratio which captures the number of women who die during pregnancy or within 42 days after ending a pregnancy, per 100,000 live births. A similar approach has been adopted by authors like [18,20,21]. Health expenditure is measured using public expenditure on health from domestic sources per capita expressed in current US dollars. Authors like [17,18,25] have equally considered a similar approach to health expenditure within extant literature. External debt is measured using total external debt stock that stand for total debt owed to non-residents repayable in currency, goods, or services in terms of current US dollars. A similar measurement approach has been employed by [26,27]. Control variables like official development aid is measured by net official development assistance per capita in disbursement flows (net of repayment of principal) that meet the DAC definition of ODA, which is in line with [28]. Foreign direct investment (FDI) is net FDI inflow (BoP, current US$) and this is similar to the measurement adopted by [29]. Finally, domestic investment is captured by gross fixed capital formation in constant 2015 US dollars, which is in line with the measurement approach of [27].

### 2.2. The Model

To achieve the objectives of the study, which is to examine public health expenditure on maternal mortality and how external debt moderates this relation, the adopted model used to examine this objective is guided by the models of [18], Refs. [20,21], which is ameliorated to suit the specifications of our study. The functional form of the empirical model is given as follows:

(1) $${MMORT}_{it}=f\left({DGGOVHLTX}_{it}^{\beta 1}+{XDBTTOT}_{it}^{\beta 2}+{HLTXDEBT}_{it}^{\beta 3}+X_{it}^{\beta j}\right)$$

where *MMORT* denotes maternal mortality, DGGOVHLTX denotes domestic government health expenditure, XDBTTOT stands for total external debt stock, and HLTXDEBT denotes the moderating term, which is the multiplication of health expenditure and total external debt, while *X* is a set of control variables. β*_j_* with *j* = 1, 2, …, *k* are the parameters of the model, and *i* and *t* are the individual and time dimensions. From the functional form of the model, we deduce the empirical model, wherein all variables are log-linearized. The empirical model can be defined as follows:

(2) $$\begin{array}{c} {LMMORT}_{it}=\beta_{0}+\beta_{1}{LDGGOVHLTX}_{it}+\;\beta_{2}{LXDBTTOT}_{it}+\beta_{3}{LHLTXDEBT}_{it}\\ +\beta_{4}{LODA}_{it}+\beta_{5}{LFDII}_{it}+\beta_{6}{LDINV}_{it}+\epsilon_{it} \end{array}$$

*LODA* denotes the log of official development aid, *LFDII* stands for log of FDI, *LDINV* is the log of domestic investment, and ϵ is the error term of the model which we assume to be normally distributed with mean zero and a constant variance. All other variables are logged and defined as in Equation (1). $\beta_{j}$ with *j* = 1, 2, …, 6 are the parameters of the model. Note that the interactive term HLTXDEBT is the product of government spending and external debt (LDGGOVHLTX∗LXDBTTOT). Taking the partial derivatives of Equation (2) with respect to public expenditure (LDGGOVHLTX) permits us to obtain the effect of debt on maternal mortality. The partial derivative obtained is given as follows:

(3) $$\frac{{\partial LMMORT}_{it}}{{\partial LDGGOVHLTX}_{it}}=\beta_{1}+\beta_{3}{LXDBTTOT}_{it}$$

The coefficients of $\beta_{1}$ and $\beta_{3}$ capture the interaction between government spending and external debt on maternal mortality. The magnitudes and signs of $\beta_{1}$ and $\beta_{3}$ depict the nature of the interaction. If $\beta_{1}$ is positive and $\beta_{3}$ is positive, it implies public expenditure increases maternal mortality and external debt is a complement to this effect by strengthening the positive effect. If $\beta_{1}$ is positive and $\beta_{3}$ is negative, it shows that government spending increases maternal mortality and external debt turns to weaken this effect. Thirdly, if $\beta_{1}$ is negative and $\beta_{3}$ is positive, it indicates that the negative effect of government spending on maternal mortality is weakened by external debt. Finally, if $\beta_{1}$ and $\beta_{3}$ are all negative, it implies that government spending reduces maternal mortality and external debt compliments this effect.

### 2.3. Empirical Model

The empirical procedure commences with a handful of diagnostic tests that guide the adoption of appropriate empirical techniques. Amidst a world wherein economic dynamics in one country often have a spillover effect on other economies, accounting for such cross-sectional dependence (CD) emanating from different events (policy contagion, regional economic shocks, and regional economic integrations) has become central in guiding empirical model adoption arguments globally [29,30]. In this regard, we employed three [31,32,33] vital CD tests to examine CD within the panel under consideration. With the establishment of CD among the panels, we proceeded to examine the data for the problems of unit root. The cross-sectional augmented Im Pesaran test (CIPS) is employed to ascertain the variables’ integration orders. Furthermore, the preliminary test requirement necessitates the examination of the panel for slope homogeneity. With CD established, the examination of the data for slope homogeneity becomes apparent [29,34]. The [35] slope homogeneity test is used in this study. Lastly, to demonstrate the presence of a long-term relationship, the study makes use of the [36,37] cointegration test approach.

Based on the different preliminary tests, we proceeded to employ the augmented mean group estimation technique proposed by [38] as an ameliorated approach to the common correlation effect mean group (CCEMG) estimator. The technique involves three key steps. Firstly, the first-difference OLS is employed to estimate a pooled regression model improved by yearly dummies. The coefficients on the (different) yearly dummies are then assembled (this stands for the common dynamic process). The estimated process of the function is added to the group-specific regression model in step two, and the regression model’s intercept captures the time-invariant fixed effects. Lastly, the model parameters relevant to each group are averaged across the panel, in the same manner as the mean group and CCEMG estimators. The AMG technique is equally good with moderate panels with both small N and T, efficiently accounting for parameter heterogeneity and CD. We further estimate the baseline model with [39] CCEMG. This approach is equally efficient due to its ability to account for CD. To further check the estimated outcome for robustness, we employed the Fixed Effect and Random Models with Driscoll and Kraay standard errors. This approach is equally second-generation in nature and robust amidst CD and panels with T greater than N. We ended the empirical procedure by employing the recently developed [40] Granger causality test to scrutinize the nature of the link between maternal mortality with the different independent variables. It is worth noting that all analyses were carried out using STATA 17 software.

## 3. Presentation of Results

Using reliable econometric estimators, the analyses in this section empirically address the controversy concerning the link between government health expenditure, external debt, and maternal mortality within the SADC zone. The descriptive and diagnostic outcomes will be presented before the empirical findings in the sections that follow.

### 3.1. Descriptive Analyses and Diagnostic Tests

The descriptive analyses and diagnostic outcomes will be presented in this section. The section presents the descriptive analyses, correlation matrix, CD test, unit root test, slope homogeneity test, and cointegration test.

#### 3.1.1. Descriptive Statistics and Correlation Matrix Outcomes

This section presents the descriptive statistics for each of the variables in the model and equally the pairwise correlation among the variables.

Table 1 presents the descriptive and the correlation outcome. The descriptive outcome shows that the average maternal mortality, government health expenditure per capita, and external debt stood at 370.553, 65.228, and 1.890 × 10^10^ with standard deviations of 184.784, 90.023, and 3.466 × 10^10^, respectively, during the period of 2000 to 2022 for the SADC economies under consideration. This shows a great dispersion above the mean for all three indicators in this study. The descriptive statistics for net ODA, net FDI, and domestic investment equally show respective averages of 54.752, 9.268 × 10^08^, and 1.021 × 10^10^. The pairwise correlation matrix demonstrates a negative correlation between all the variables of the model and maternal mortality. However, the correlation between the state health spending and the interactive terms is relatively stronger.

#### 3.1.2. Cross-Sectional Dependence

To account for key biases in the estimation techniques adopted, the diagnostics tests employed in this study commence with the CD test, which permits us to make an appropriate choice of the estimation technique.

The [31,32,33] test reported in Table 2 illustrates that the Friedman and Pesaran tests reject the null hypothesis that there is no CD at the 1% level of significance. Additionally, according to the test critical values of the Frees test, the null hypothesis that there is no CD is similarly rejected at the 1% level. This shows strong evidence of interdependence among the economies of the SADC zone under consideration, and, as such, the adoption of appropriate econometric techniques that account for such interdependence becomes imminent.

#### 3.1.3. Unit Root Test Outcome

After the CD test, we proceed with the presentation of the panel unit root test using the CIPS test.

The outcome of the CIPS unit root test in Table 3 presents both the outcome with and without a trend. The test result shows that maternal mortality and government expenditures show the null hypotheses of homogenous nonstationary behavior for maternal mortality and government spending is rejected at both levels, with trend and without trend, since their respective test statistics lie above the critical value of −2.25 (the case without trend) and −2.76 (the case with trend). This shows that these variables follow an I(0) process. With regard to external debt, the null hypothesis of homogenous nonstationary behavior is not refuted at the level. However, at the first difference, it becomes stationary since both the test statistics with trend and without trend of −3.684 and −3.986, respectively, all lie above the 1% critical value of −2.45 (without trend) and −2.96 (with trend). This implies that external debt follows an I(1) process and is stationary at first difference. With regard to the control variables, the interactive term, ODA, and FDI are all stationary at level. Domestic investment on its part only becomes stationary for the test without trend and with trend at I(1).

#### 3.1.4. Slope Homogeneity and Cointegration Test Outcomes

The panel is further examined for slope homogeneity and cointegration. The results of the slope homogeneity and cointegration tests are shown in Table 4. The results of the slope homogeneity test demonstrate that, at a 1% significant level, the null hypothesis of homogeneous slopes is rejected for both the delta and modified delta test statistics.

This implies that all slope coefficients are not identical across cross-sectional units, confirming slope heterogeneity. As explained earlier, Refs. [36,37] are employed to establish the existence of a long-run relation. The outcome reveals that the Modified Phillips–Perron, Phillips–Perron, and Augmented Dickey–Fuller statistics from the Pedroni test all reject the null hypothesis of no cointegration at 1%, 1%, and 10%, respectively. Similarly, at the 1% level of significance, the Westerlund test statistics for the variance ratio test indicate that the null hypothesis of no cointegration is likewise rejected. This confirmed that the panel of 13 SADC economies under examination had a long-term relationship.

### 3.2. The Estimated Outcomes

This section presents the estimation outcome of the baseline model AMG and the CCEMG techniques. The robustness of the outcome is then examine using the Driscoll–Kraay (DK) fixed and random effect techniques, and, finally, the variables are examined for causality.

#### 3.2.1. AMG and CCEMG Results

With the establishment of cointegration and the existence of CD, we adopt the augmented mean group (AMG) and the common correlation effect mean group (CCEMG) estimation technique to estimate our baseline model.

The outcome of the two estimated techniques adopted for the baseline model presented in Table 5 shows a consensus that the government’s health expenditure decreases maternal mortality within the SADC sub-region. The results further reveal that external debt curbs maternal mortality within the SADC region. The interactive term is significantly positive for the AMG and CCEMG models. This shows that the negative effects of the government health expenditure on maternal mortality are mitigated and decreased by external debt.

Official development aid, foreign direct investment, and domestic investment act as curbing agents for maternal mortality. The result shows that these variables are negatively related to maternal mortality; that is, an increase in official development aid, foreign direct investment, and domestic investment leads to a reduction in maternal mortality for the SADC economies under consideration. This is eminent, given that these variables boost the capital structure of these economies and enhance the capitalization of the health sector, which leads to the provision of better health that translates into a reduction in maternal mortality. With regard to the fitness of our models for inference, the Wald test statistics for both the AMG and CCEMG estimates are all significant at 1%, showing the global fitness of the model. Equally, the low value of the Root Mean Squared Error of 0.0488 and 0.0371 for the AMG and CCEMG models, respectively, shows that there is better model performance since it indicates that the predictions are closer to the actual value. Finally, the common dynamic process (CDP) value is significant, indicating that there are common dynamic factors affecting all units in the panel under consideration.

#### 3.2.2. Robustness Check

We further checked our models more for robustness, by estimating the models using the Driscoll–Kraay (DK) and fixed and random techniques. This was carried out primarily to show that the outcome is consistent across different estimation techniques.

The outcome of the DK model is presented in Table 6. The results reveal, consistent with the AMG and the CCEMG estimates, that the government expenditure leads to a positive effect on maternal mortality. This outcome is obtained for both the DK fixed effect and the DK random effect estimates. In the same vein, the negative effect of the external debt stock on maternal mortality is reaffirmed for both the DK fixed effect and the DK random effect estimates. This shows that, with variations of the estimation approach, the established results of the external debt stock and government’s health expenditure still reduce maternal mortality within the SADC sub-region, although with variations in the degree of significance of the different models. The moderating term is positive and significant for both the DK fixed effect and the DK random effect estimates. This confirms that the government’s health expenditure on maternal mortality in southern Africa is less effective as external debt is accounted for. Furthermore, the DK fixed effect and the DK random effect estimates verify the negative effects of official development aid, foreign direct investment, and domestic investment. However, the DK random effect model does not find any significance for official development aid. The significant values of the F statistics for the DK fixed effect model and the Chi2 statistics for the DK random effect model demonstrate the result’s global fitness

#### 3.2.3. Causality Test Result

The Granger causality test by [40] is also utilized in this study to examine the causal relationship between the model’s various variables. The causality test outcome (presented in Table 7) reveals that the null hypotheses of government health spending not granger-causing maternal mortality and maternal mortality not granger-causing government health expenditure are all rejected at the 1% level of significance.

This demonstrates that, for the SADC economies under study, there is a bidirectional relationship between the government’s health spending and maternal mortality. Similarly, the null hypotheses of external debt not granger-causing maternal mortality and maternal mortality not granger-causing external debt are rejected at the 1% level of significance. This equally establishes a bidirectional relationship between the external debt stock and maternal mortality. The outcome equally establishes a unidirectional causality from the interactive term to maternal mortality. With regard to the control variables, a bidirectional relationship is equally observed between maternal mortality and foreign direct investment, and maternal mortality and domestic investment. However, official development demonstrates a unidirectional relationship from official development aid to maternal mortality.

## 4. Discussion of Key Findings

This outcome aligns with the extant theoretical and empirical literature that argues that health spending reduces maternal mortality within economies of the world [9,10,13]. This shows that the effort employed by the governments of the SADC economies to boost health services within their respective economies is yielding positive outcomes on maternal health. This is in line with the discussion of [12], which highlighted that a reduction in government health spending can generate higher maternal deaths through a reduction of health personnel and the inability to equip health facilities with health personnel. Similarly, the results buttress the view of [41], which argues that a major improvement in healthcare coverage can save a substantial number of maternal and newborn fatalities, especially in low- to middle-Human Development Index (HDI) nations. However, it is critical to understand that the effectiveness of healthcare spending is determined by how the money is allocated. For example, health investments must be deliberately directed into maternal health initiatives to reap large returns. This has equally led authors like [15] to argue, within Asian economies, that, without the ample allocation of health spending, it will lead to an increase in maternal mortality.

The findings revealed that external debt reduces maternal mortality within the SADC regions. This result observed is contrary to [20,21,22], which decided that debt increases maternal mortality. The negative impact of external debt on maternal mortality can be explained in line with the views that a low level of debt can enhance economic performance. Stylized facts presented by [7] show that, during the study period, Southern economies generally reduced their external debt more than any other sub-regional grouping within SSA.

The moderation investigation in our study showed that external debt moderates and reduces the effect of the government health expenditure on maternal mortality. This is evident given the high levels of debts in economies like South Africa, Angola, and Mozambique (see Figure 3), which tend to require higher debt servicing amounts, which reduces available funds by the state to spend on health services. This can equally tend to affirm the perspective of Ibohm and Feraru [19], who discuss that debt relief reduces maternal mortality. This will further imply that controlling the level of debt can lead to better health conditions that tend to reduce maternal mortality within the SADC sub-region.

The causal analyses equally provided vital insight into the nature of the link between the key dependent variables (government expenditure and external debt) and independent variable. The bidirectional relationship between maternal mortality and government expenditure shows that information about the future path of maternal mortality is contained in government expenditure, and information about future government health expenditure is equally contained in maternal mortality. This implies that health spending and mortality are interdependent within the SADC region. External debt evolution is equally determined by maternal mortality and this relationship is seen equally in both directions.

## 5. Conclusions

The impact of external debt on maternal mortality and the government’s health spending have been the subject of ongoing theoretical and empirical discussions. But the relationship between health expenditures, foreign debt, and maternal mortality has remained contentious, with no clear understanding of how external debt influences the relationship between the government’s health expenditure and maternal death. Our study contributes to the existing literature by examining the moderating role of external debt on the government’s health expenditure and maternal mortality relation using the data of 13 SADC economies. We adopt the augmented mean group, common correlation effect mean group, the Driscoll/Kraay technique, and the Juodis et al. [40] Granger causality test which are robust to CD, heterogeneity, serial correlation, and heteroscedasticity. Our findings revealed that the government’s health expenditure and external debt reduce maternal mortality in the SADC region. Equally, the magnitude of the government’s health spending is moderated by the external debt stock. We argue that increasing the level of health expenditure enhances the health sectors of developing economies like those of the SADC region through increased health personnel, and the purchase of up-to-date medical equipment among others, leading to better health services that reduce maternal mortality. The outcome equally establishes a bidirectional relation between external debt and maternal mortality and equally between maternal mortality and public health spending.

About policy recommendations, with regard to the negative effect of health spending on maternal mortality, we recommend that more financial resources be channeled towards the health sector by policymakers within the SADC zone to boost health provisions which will translate to a reduction in maternal deaths and better healthcare. Equally, regarding external debt, when domestic actors contract debt, investment in the health sector, like the construction of health infrastructure, the purchase of medical equipment, and the training of health personnel, is recommended since this will enhance the healthcare system and reduce maternal mortality. Finally, it is worth noting that, although seeking external finance like external debt is productive for the health sector, care should be taken not to acquire excessive debt that will increase the debt burden and tend to partial out the effect of the government’s health expenditure on maternal mortality.

This current study equally has some key limitations. Notably, the number of countries within the SADC region considered in this study is limited to 13 and the study period is limited for the period of 2000 to 2022. Further studies can be carried out with a larger time period and an increase in the number of countries. Equally, country-by-country analyses are not considered in this study. This could permit country-specific recommendations and provide grounds for more comparisons. In this regard, further studies for specific countries within SADC can be considered. Equally, other estimation techniques like the dynamic common correlation effect and the panel smooth transition technique can be considered by others to examine the validity of the current outcome and examine short- and long-run dynamics.

## Figures and Tables

**Table 1:** Descriptive statistics and correlation matrix.

**Descriptive Statistics**										
Variable	Obs		Mean		Std. Dev.		Min		Max	
MMORT	299		370.553		184.784		42		860	
DGGOVHLTX	299		65.228		90.023		0.176		360.01	
XDBTTOT	276		1.890 × 10^10^		3.466 × 10^10^		1.256 × 10^08^		1.907 × 10^11^	
ODA	299		54.752		37.818		−11.967		264.705	
FDII	299		9.268 × 10^08^		3.035 × 10^09^		−7.397 × 10^09^		4.066 × 10^10^	
DINV	231		1.021 × 10^10^		1.604 × 10^10^		70,766,640		6.244 × 10^10^	
**Pairwise Correlations**										
Variables	(1)	(2)		(3)		(4)	(5)	(6)		(7)
(1) LMMORT	1.000									
(2) LDGGOVHLTX	−0.534 *** (0.000)	1.000								
(3) LXDBTTOT	−0.291 *** (0.000)	0.303 *** (0.000)		1.000						
(4) LHLTXDEBT	−0.618 *** (0.000)	0.769 *** (0.000)		0.520 *** (0.000)		1.000				
(5) LODA	−0.050 (0.392)	−0.025 (0.673)		−0.419 *** (0.000)		−0.247 *** (0.000)	1.000			
(6) LFDII	−0.052 (0.368)	0.057 (0.327)		0.082 (0.174)		0.086 (0.153)	0.219 *** (0.000)	1.000		
(7) LDINV	−0.140 ** (0.034)	0.228 *** (0.001)		0.912 *** (0.000)		0.483 *** (0.000)	−0.526 *** (0.000)	0.061 (0.354)		1.000

*p* values in parentheses; *** and ** denote 1% and 5% significant levels, respectively.

**Table 2:** CD test outcome.

Test	Test Statistics	Critical Values/*p*-Values
Frees test	3.150	[0.2225, 0.1537, 0.1174]
Friedman test	79.921	0.0000
Pesaran 2015 test	10.592	0.0000

**Table 3:** CIPS unit root test.

Variables	CIPS without Trend	CIPS with Trend	Decision
	Test Statistics	Test Statistics	
LMMORT	−2.400 **	−2.994 ***	I(0)
LDGGOVHLTX	−2.418 **	−3.067 ***	I(0)
LXDBTTOT	−0.943	−1.633	
D(LXDBTTOT)	−3.684 ***	−3.986 ***	I(1)
LHLTXDEBT	−2.207 *	−2.999 ***	I(0)
LODA	−2.677 ***	−2.673 **	I(0)
LFDII	−2.641 ***	−3.098 ***	I(0)
LDINV	−2.200 *	−2.309	
D(LDINV)	−4.278 ***	−4.739 ***	I(1)

Critical values (without trend (−2.14 (10%), −2.25 (5%), and −2.45 (1%)), and with trend (−2.66 (10%), −2.76 (5%), and −2.96 (1%))). *, **, and *** indicate 10%, 5%, and 1% significance.

**Table 4:** Slope homogeneity and cointegration test outcome.

Test Statistics	Coefficients	*p* Value
**Slope homogeneity**		
Delta	11.211	0.000
Adjusted Delta	13.452	0.000
**Cointegration test**		
**Pedroni Cointegration test**		
Modified Phillips–Perron t	2.8548	0.0022
Phillips–Perron t	−3.4035	0.0003
Augmented Dickey–Fuller t	−1.3385	0.0904
**Westerlund Cointegration Test**		
Variance ratio	3.6610	0.0001

**Table 5:** Baseline regression (AMG and CCE regression).

VARIABLES LMMORT	(1)	(2)
	AMG	CCE
LDGGOVHLTX	−4.285 **	−2.689 *
	(1.976)	(1.595)
LXDBTTOT	−0.747 ***	−0.512 **
	(0.137)	(0.258)
LHLTXDEBT	0.204 **	0.132 *
	(0.0817)	(0.0759)
LODA	−0.0232	−0.0223
	(0.0258)	(0.0352)
LFDII	−0.0149	−8.915
	(0.178)	(9.002)
LDINV	−0.207 *	−0.269 **
	(0.107)	(0.127)
c_d_p	1.210 ***	
	(0.263)	
Constant	22.84	210.7
	(18.43)	(207.2)
Wald chi2(6)	45.18 *** [0.0000]	13.32 ** [0.0382]
RMSE(sigma)	0.0488	0.0371
Observations	206	206
Number of COUNTY	9	9

Standard errors in parentheses. *** *p* < 0.01, ** *p* < 0.05, * *p* < 0.1.

**Table 6:** Robustness check (DK regression).

	(1)	(2)
VARIABLES	FE	RE
LDGGOVHLTX	−1.146 *	−1.164 **
	(0.564)	(0.501)
LXDBTTOT	−0.459 ***	−0.421 ***
	(0.0648)	(0.0594)
LHLTXDEBT	0.0527 **	0.0512 **
	(0.0215)	(0.0186)
LODA	−0.0628 ***	−0.0537
	(0.0209)	(0.0341)
LFDII	−0.0515 *	−0.0599 *
	(0.0296)	(0.0344)
LDINV	−0.288 ***	−0.254 ***
	(0.0224)	(0.0492)
Constant	20.78 ***	19.17 ***
	(1.441)	(1.268)
Observations	207	207
Number of groups	10	10
F	1049 [0.000]	
chi2		2537 [0.000]

Standard errors in parentheses. *** *p* < 0.01, ** *p* < 0.05, * *p* < 0.1.

**Table 7:** Causality test.

Hypothesis	Test Statistics	*p* Value
LMMORT # LDGGOVHLTX	44.532	0.0000
LDGGOVHLTX # LMMORT	7.00093	0.0081
LMMORT # LXDBTTOT	14.0579	0.0000
LXDBTTOT # LMMORT	17.514015	0.0009
LMMORT # LHLTXDEBT	40.317357	0.0000
LHLTXDEBT # LMMORT	1.2010414	0.2731
LMMORT # LODA	5.4072236	0.0670
LODA # LMMORT	2.6687932	0.1023
LMMORT # LFDII	4.9217085	0.0854
LFDII # LMMORT	8.0482953	0.0046
LMMORT # LDINV	29.849062	0.0000
LDINV # LMMORT	6.263586	0.0123

# = does not granger cause.

## Data Availability

Data are contained within the article.
